# Stimulatory and inhibitory effects of phenanthrene on physiological performance of *Chlorella vulgaris* and *Skeletonema costatum*

**DOI:** 10.1038/s41598-022-08733-9

**Published:** 2022-03-25

**Authors:** Lele Jiang, Yueqiang Pan, Shaoting Zhu, Jingmin Qiu, Yu Shang, Juntian Xu, Futian Li, Hongbin Wang

**Affiliations:** 1 Jiangsu Key Laboratory of Marine Bioresources and Environment, Jiangsu Ocean University, Lianyungang, 222005 China; 2 Marine Resources Development Institute of Jiangsu, Jiangsu Ocean University, Lianyungang, 222005 China

**Keywords:** Environmental impact, Marine biology

## Abstract

The effects of polycyclic aromatic hydrocarbons on phytoplankton have been extensively documented, but there is limited knowledge about the physiological responses of marine primary producers to phenanthrene at environmentally relevant levels. Here, we investigated the toxicity of phenanthrene (0, 1, and 5 or 10 μg L^−1^) to the physiological performance of two cosmopolitan phytoplankton species: the green alga *Chlorella vulgaris* and bloom-forming diatom *Skeletonema costatum*. The specific growth rates of both species were remarkably inhibited at both low (1 μg L^−1^) and high phenanthrene concentrations (5 or 10 μg L^−1^), while their tolerance to phenanthrene differed. At the highest phenanthrene concentration (10 μg L^−1^), the growth of *C. vulgaris* was inhibited by 69%, and no growth was observed for *S. costatum* cells. The superoxide dismutase activity of both species was enhanced at high phenanthrene concentration, and increased activity of catalase was only observed at high phenanthrene concentration in *C. vulgaris*. Interestingly, the low phenanthrene concentration stimulated the photosynthetic and relative electron transport rates of *S. costatum*, whereas hormetic effects were not found for growth. Based on our results, phenanthrene could be detrimental to these two species at a environmentally relevant level, while different tolerance levels were detected.

## Introduction

Polyaromatic aromatic hydrocarbons (PAHs) are organic pollutants resulted from pyrogenic and petrogenic sources^1^. Phenanthrene is a three-ring PAH that has frequently been chosen as a representative PAH to investigate their influences and biodegradation^2^. With a stable chemical structure, phenanthrene can be adsorbed on suspended solids, microplastics, and sediments in water for long-distance migration^3^. Phenanthrene concentrations in the River Tees in England and Daya Bay in China were higher than 1 μg L^−1^^4,5^. The phenanthrene concentrations in sediment pore water from the Jiulong River and Western Xiamen Sea ranged from 2.4 to 26.1 μg L^−1^^6^. Existing studies have shown that phenanthrene is potentially toxic to aquatic life, such as fish, shellfish, phytoplankton, and aquatic insects, and higher phenanthrene concentrations than environmental values have been reported in certain organisms^7–9^.

Marine microalgae have attracted much attention as the base of marine food webs. Microalgae are sensitive to pollutants and can be used as indicator organisms. The potential impacts of exogenous pollutants on the whole ecological environment can be reflected by studying and measuring their effects on the growth of microalgae^10^. The influences of PAHs on phytoplankton have been extensively investigated. The toxicity of phenanthrene to *Chlorella salina* was shown to be influenced by culture pH, with decreased concentrations required to inhibit growth by 50% as the pH decreased^11^. The toxicity of PAHs also depends on the exposure time. Short-term (24 h) exposure to benzo [k] fluoranthene showed no effects on the growth of *C.* *vulgaris*, while the inhibitory effect became more obvious after 48 h with the increase of benzene [k] fluoranthene concentration^12^. Phytoplankton exhibited species-specific tolerance to PAH toxicity^13^, which could alter community succession^14^. The antioxidant defense system plays a crucial role in protecting cells from oxidative stresses. Environmental stressors, such as chemical pollution, disturb the homeostasis of intracellular pro-oxidants and antioxidants, and high pollution levels damage the antioxidant defense system^15^.

An increasing number of studies have investigated the effects of contaminants because of the increasing severity of pollution in the marine environment. However, there is limited knowledge about the effects of environmentally relevant levels of PAHs on typical phytoplankton species. Here, we investigated the toxicity of phenanthrene, a representative PAH, to the marine green alga *C. vulgaris* and the bloom-forming diatom *Skeletonema costatum*. Physiological characteristics, including growth, pigment, photosynthesis and dark respiration rates, chlorophyll fluorescence parameters, and superoxide dismutase and catalase activity, were measured to provide a comprehensive evaluation of phenanthrene toxicity.

## Results

### Specific growth rate

**Figure 1:**
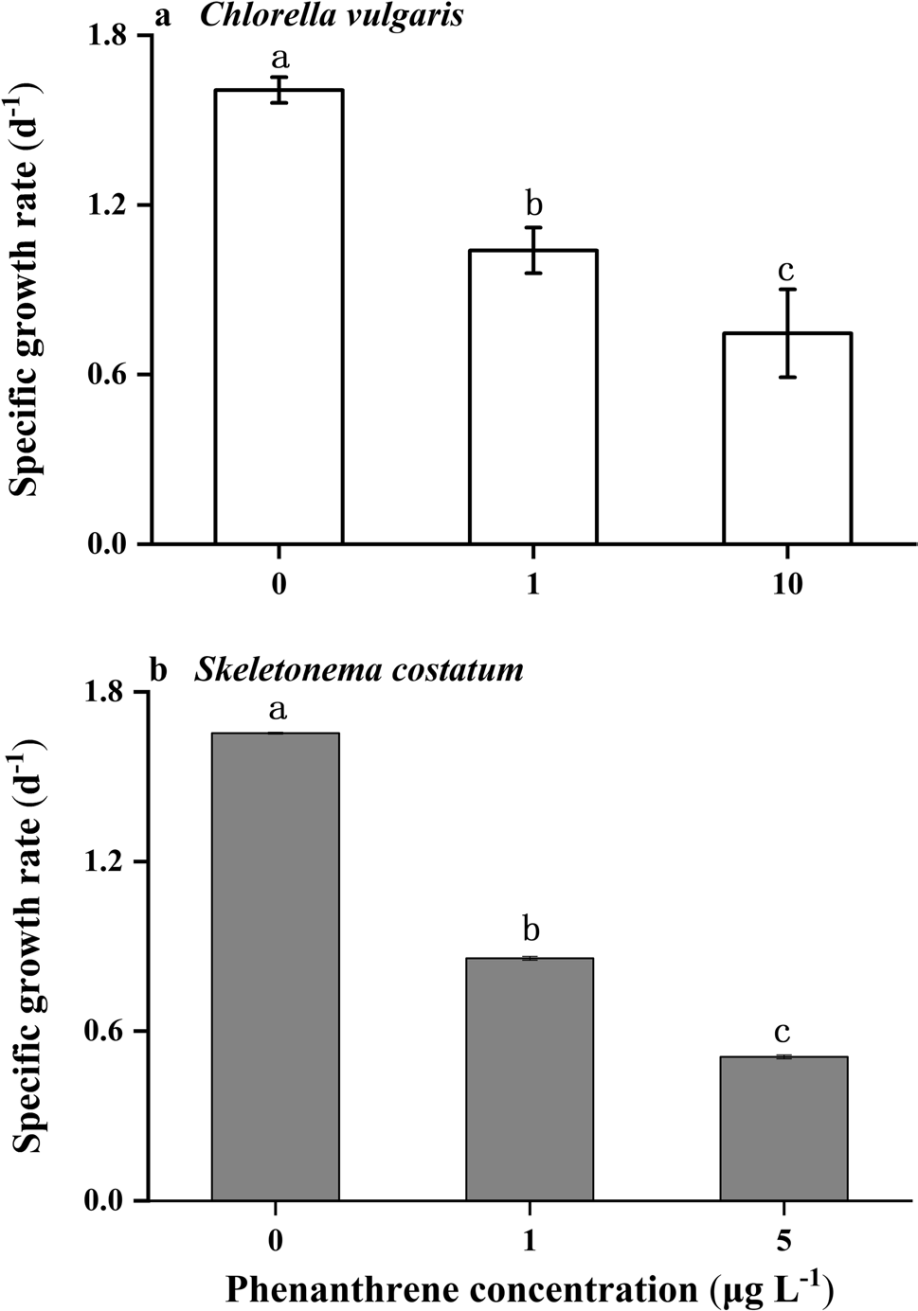
The specific growth rates (d^−1^) of *Chlorella vulgaris* **(a)** and *Skeletonema costatum* **(b)** cells at different phenanthrene concentrations. The data are means ± SD of triplicate cultures (n = 3). The different lowercase letters indicate significant (*p* < 0.05) differences between phenanthrene concentrations in one species (one-way ANOVA and post-hoc Tukey tests).

Different concentrations of phenanthrene had significant effects on the specific growth rates of *C. vulgaris* and *S. costatum* (Fig. 1, *p* < 0.001). The specific growth rates of *C. vulgaris* at the low and high concentrations of phenanthrene were 35% and 53% lower than the control, respectively. In contrast, the specific growth rates of *S. costatum* at the low and high concentrations of phenanthrene were 48% and 69% lower than the control, respectively.

### Chlorophyll *a* and carotenoid contents

**Figure 2:**
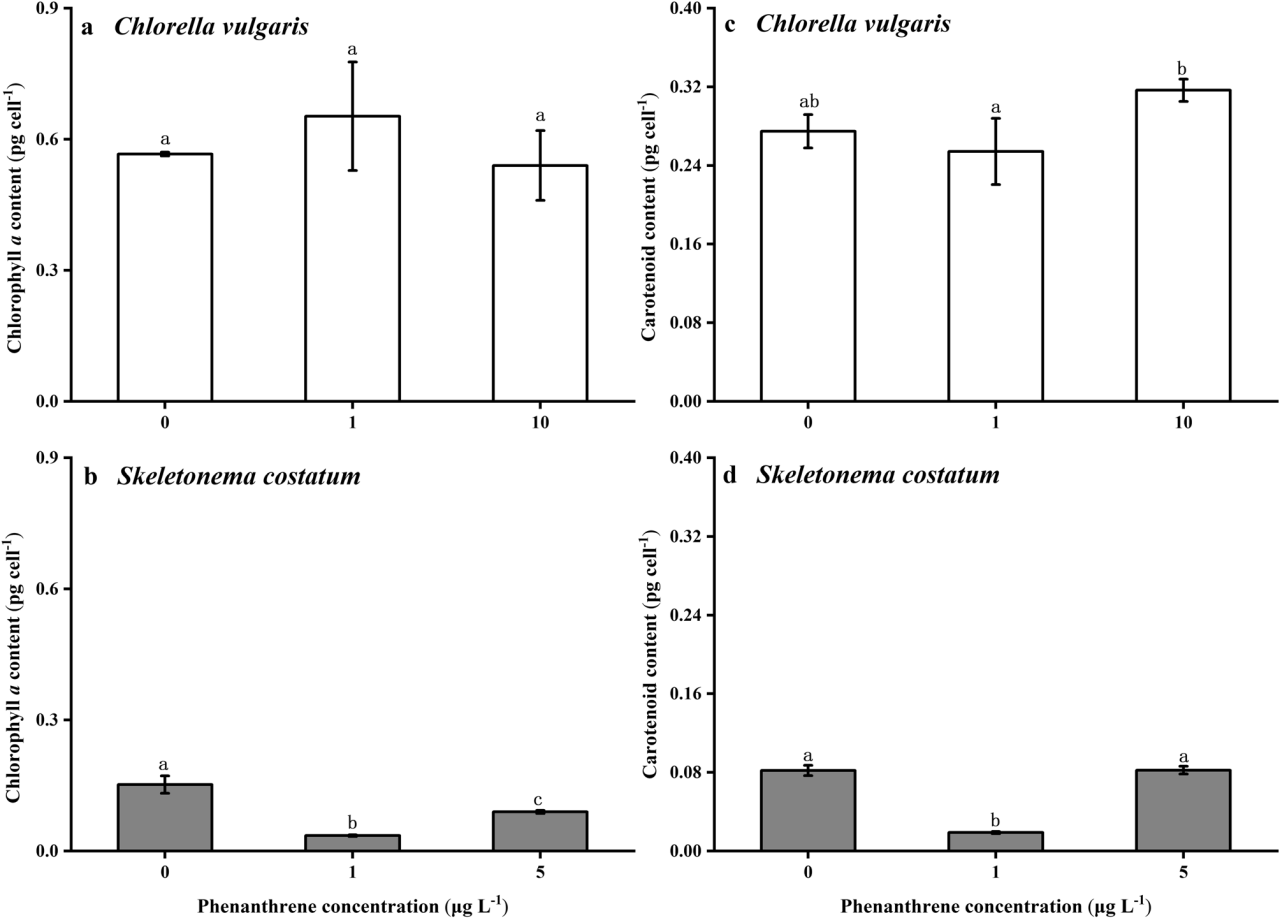
Cellular quotas of chlorophyll *a* **(a,b)** and carotenoid content **(c,d)** (pg cell^−1^) of *Chlorella vulgaris* **(a,c)** and *Skeletonema costatum* **(b,d)** cells at different phenanthrene concentrations. The data are means ± SD of triplicate cultures (n = 3). The different lowercase letters indicate significant (*p* < 0.05) differences between phenanthrene concentrations in one species (one-way ANOVA and post-hoc Tukey tests).

Phenanthrene showed no significant adverse effects on the chlorophyll *a* and carotenoid contents of *C. vulgaris* (Fig. 2a,c). The contents of chlorophyll *a* of *S. costatum* were 77% and 61% lower than the control at the low and high phenanthrene concentrations, respectively (Fig. 2b). The carotenoid content of *S. costatum* at the low concentration of phenanthrene was 77% lower than the control and high phenanthrene concentration (Fig. 2d). Phenanthrene showed significant effects on the chlorophyll *a* and carotenoid contents of *S. costatum* (*p* < 0.001).

### Chlorophyll fluorescence

**Figure 3:**
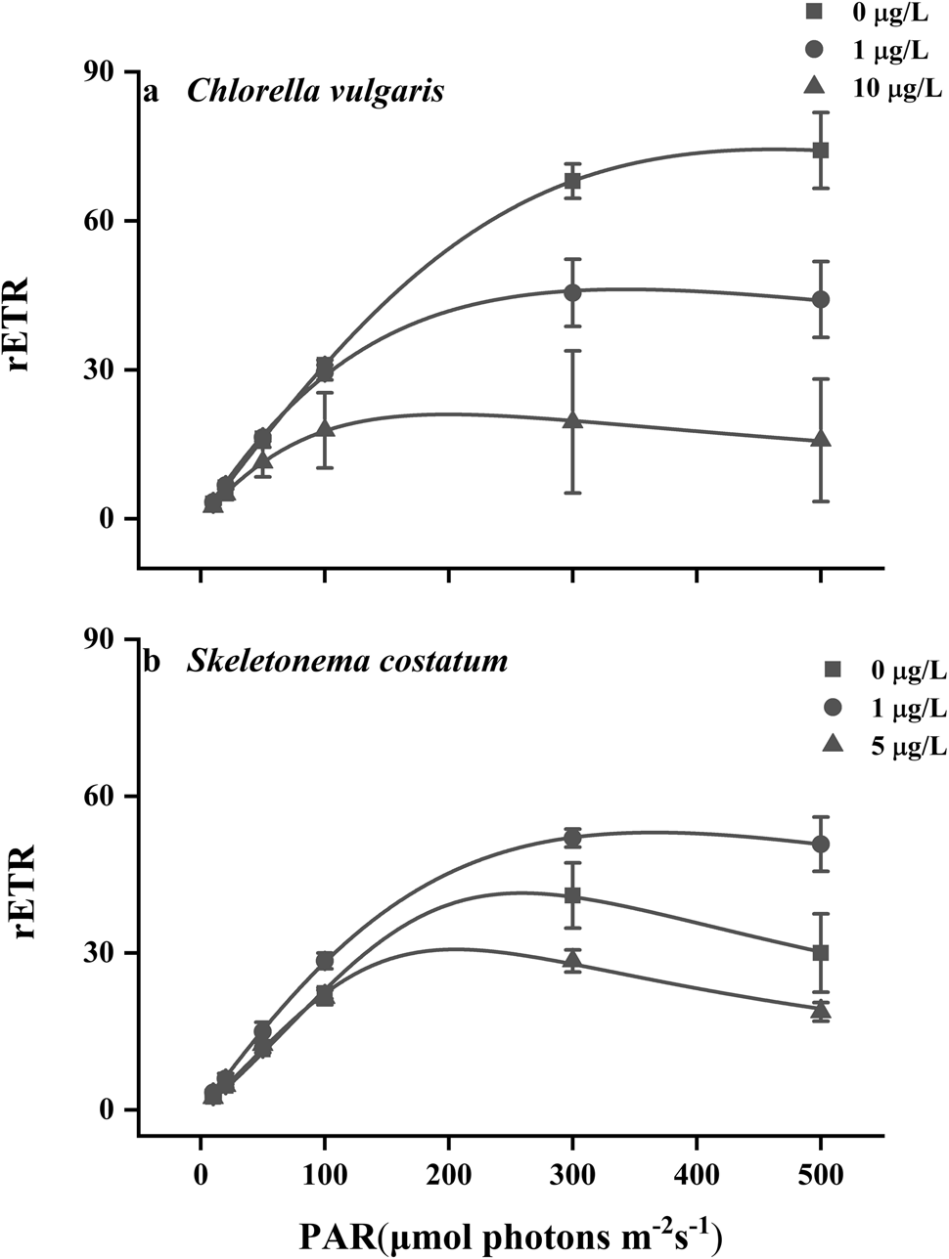
The rapid light curves determined by variations of relative electron transport rate (rETR) under progressively increasing light intensities in *Chlorella vulgaris* **(a)** and *Skeletonema costatum* **(b)** cells at different phenanthrene concentrations. The data are means ± SD of triplicate cultures (n = 3).

The low and high concentrations of phenanthrene showed negative influences on the rapid light curves (RLCs) of *C. vulgaris* cells (Fig. 3a), which was reflected by the depressed maximum relative electron transport rate (rETR_max_) and light saturation point (I_k_) (Table 1). There was no significant difference in the value of α among treatments. In comparison, the effects of phenanthrene on the chlorophyll fluorescence parameters of *S. costatum* depended on its concentration (Fig. 3b). The low concentration of phenanthrene enhanced the rETR_max_ and α values of *S. costatum* cells by 26% and 68%, respectively (Table 1). However, *S. costatum* cells exposed to the high phenanthrene concentration showed decreased rETR_max_ and I_k_. (Table 1).

**Table 1:** The maximum relative electron transport rate (rETR_max_), apparent photon transfer efficiency (α), and light saturation point (I_k_) (μmol photons m^−2^ s^−1^) of *Chlorella vulgaris* and *Skeletonema costatum* cells at different phenanthrene concentrations.

Species	Phenanthrene concentration (μg L^−1^)	rETR_max_	α	I_k_ (μmol photons m^−2^ s^−1^)
*Chlorella vulgaris*	0	75 ± 7^a^	0.32 ± 0.01^ab^	330 ± 12^a^
	1	46 ± 7^b^	0.38 ± 0.01^a^	121 ± 21^b^
	10	22 ± 13^c^	0.28 ± 0.05^b^	76 ± 34^b^
*Skeletonema costatum*	0	42 ± 5^a^	0.19 ± 0.02^a^	219 ± 16^a^
	1	53 ± 2^b^	0.32 ± 0.06^b^	169 ± 27^ab^
	5	31 ± 3^a^	0.21 ± 0.01^ab^	143 ± 11^b^

The different superscript letters indicate significant (*p* < 0.05) differences between phenanthrene concentrations in one species (one-way ANOVA and post-hoc Tukey tests).

### Photosynthesis and dark respiration

**Figure 4:**
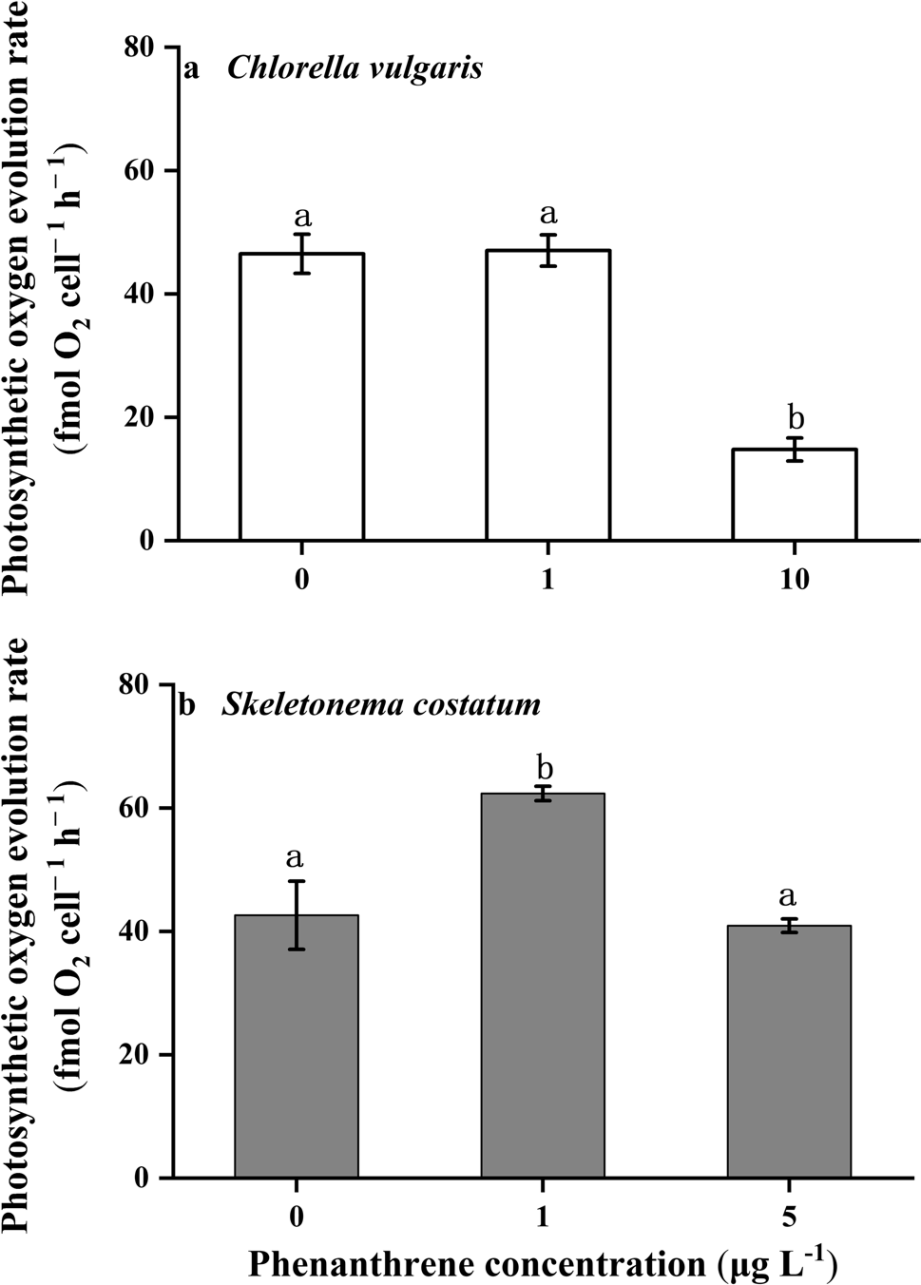
The photosynthesis rates of *Chlorella vulgaris* **(a)** and *Skeletonema costatum* **(b)** cells at different phenanthrene concentrations. The data are means ± SD of triplicate cultures (n = 3). The different lowercase letters indicate significant (*p* < 0.05) differences between phenanthrene concentrations in one species (one-way ANOVA and post-hoc Tukey tests).

**Figure 5:**
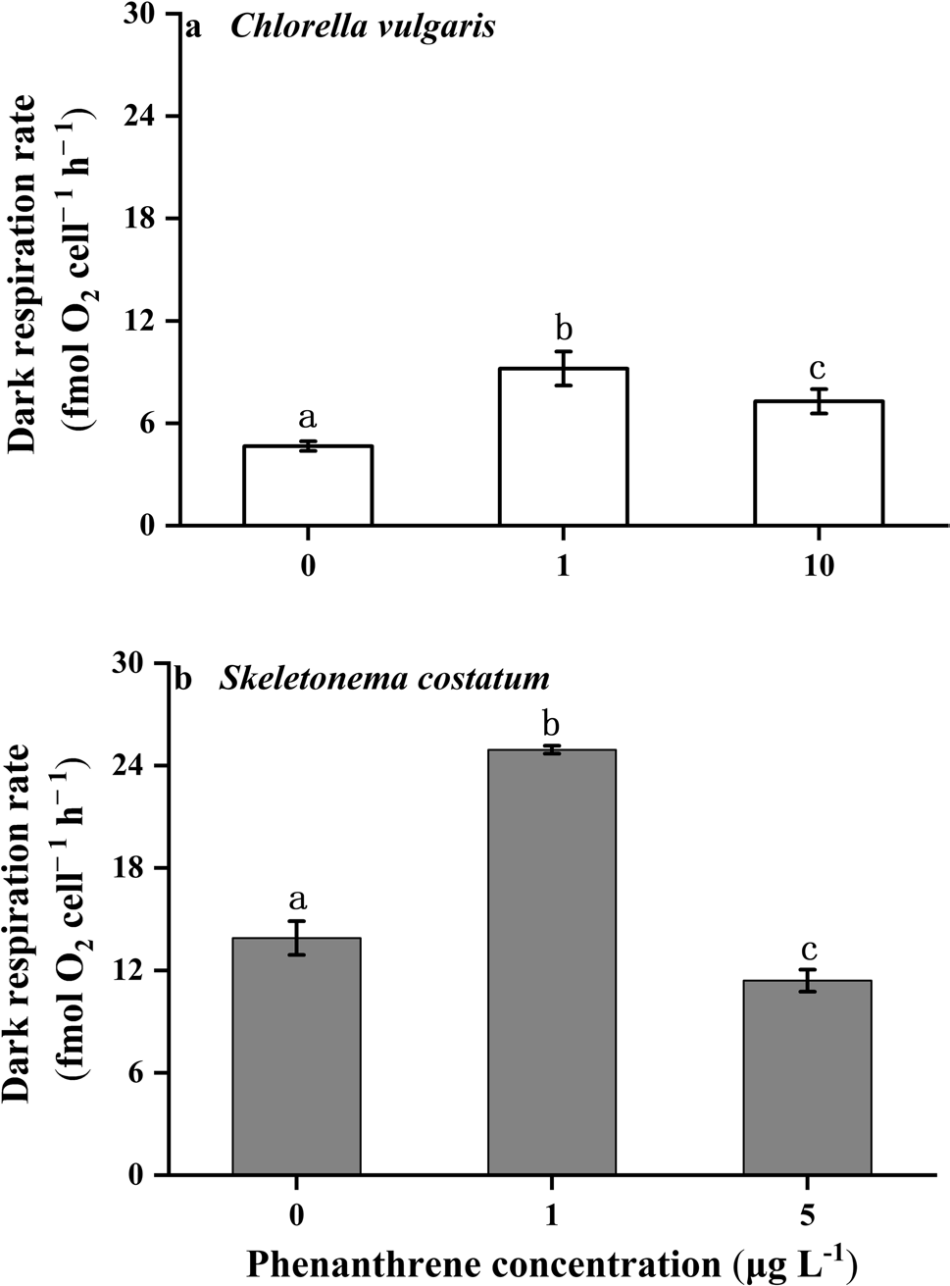
The dark respiration rates of *Chlorella vulgaris* **(a)** and *Skeletonema costatum* **(b)** cells at different phenanthrene concentrations. The data are means ± SD of triplicate cultures (n = 3). The different lowercase letters indicate significant (*p* < 0.05) differences between phenanthrene concentrations in one species (one-way ANOVA and post-hoc Tukey tests).

The phenanthrene showed obvious influences on the photosynthetic oxygen evolution rates of *C. vulgaris* and *S. costatum* (*p* = 0.002 and *p* < 0.001, respectively). The photosynthetic oxygen evolution rate of *C. vulgaris* at the high concentration of phenanthrene was the lowest, which was lower than the control (Fig. 4a). The photosynthetic oxygen evolution rate of *S. costatum* at the low concentration of phenanthrene was the highest, which was 46% and 52% higher than the control and high phenanthrene concentrations, respectively (Fig. 4b). The dark respiration rates of the two species were the highest under the low concentration of phenanthrene, which indicated that the low concentration of phenanthrene promoted the dark respiration rates (Fig. 5).

### Superoxide dismutase and catalase activity

**Figure 6:**
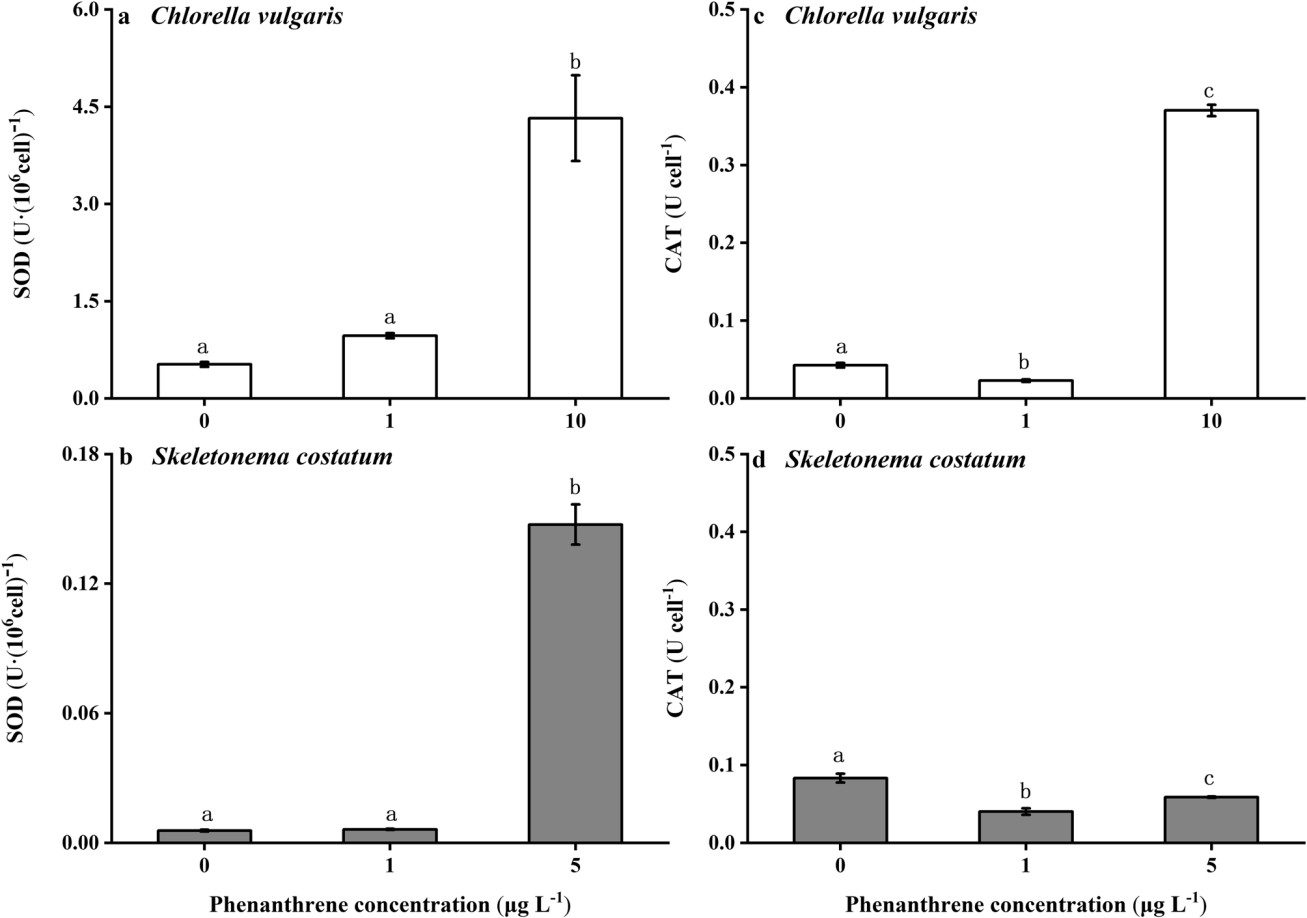
The superoxide dismutase (SOD) **(a,b)** and catalase (CAT) **(c,d)** activity of *Chlorella vulgaris* **(a,c)** and *Skeletonema costatum* **(b,d)** cells at different phenanthrene concentrations. The data are means ± SD of triplicate cultures (n = 3). The different lowercase letters indicate significant (*p* < 0.05) differences between phenanthrene concentrations in one species (one-way ANOVA and post-hoc Tukey tests).

The high phenanthrene concentrations showed significant effects on the superoxide (SOD) activity of both species (Fig. 6, *p* < 0.001). The SOD activity of *C. vulgaris* and *S. costatum* was highest under the high phenanthrene concentration. The SOD activity of *C. vulgaris* at the high phenanthrene concentration was eight and five times higher than that at the control and the low phenanthrene concentration, respectively. The SOD activity of *S. costatum* at the high phenanthrene concentration was 26 and 24 times higher than that at the control and the low phenanthrene concentration, respectively. The catalase (CAT) activity of *C. vulgaris* at the high phenanthrene concentration was 9 and 16 times higher than that at the control and the low phenanthrene concentration, respectively (Fig. 6c, *p* < 0.05). The lowest CAT activity of *S. costatum* was found at the low phenanthrene concentration (Fig. 6d, *p* < 0.001).

## Discussion

Marine *C. vulgaris* and *S. costatum* belong to two key phytoplankton groups, and they are usually used as biological indicators of marine environmental monitoring. In the present study, we exposed these two species to three phenanthrene concentrations, which might be experienced by phytoplankton in marine environments. The specific growth rates of two species were significantly inhibited at low and high phenanthrene concentrations, while their toxicity sensitivity differed. The low concentration of phenanthrene promoted the photosynthetic and electron transport rates of the *S. costatum*, while the high concentration resulted in significant inhibition.

The two species grew similarly under the control condition, whereas the growth rates of *S. costatum* were generally lower than those of *C. vulgaris* at low and high phenanthrene concentrations. *S. costatum* cells could not grow when they were exposed to 10 μg L^−1^ of phenanthrene. In contrast, the growth rate of *C. vulgaris* at 10 μg L^−1^ phenanthrene was still 32% higher than that of *S. costatum* cells treated with 5 μg L^−1^ phenanthrene. This may have been because the higher growth rate of *C. vulgaris* led to higher cell density, thus the adsorption of phenanthrene per cell decreased relative to that at a lower cell density^13^. It has been suggested that the toxicity tolerance of different species may depend on the ratio of the surface area to the volume and lipid content of the cell membrane^16,17^. *S. costatum* cells used in the present study were in unicellular forms and the two species had similar cell size and lipid productivity^18,19^. Thus, other mechanisms might have been responsible for the different toxicity tolerance. In the present study, we found that the SOD and CAT activity of *C. vulgaris* was markedly higher than that of *S. costatum*, which could have led to the higher tolerance of the green alga to phenanthrene.

Decreased growth and total biomass were observed when *C. vulgaris* was exposed to phenanthrene concentrations of 1 and 10 μg L^−1^ for 72 h, and the negative effects were increasingly obvious as the phenanthrene concentrations increased^8^. This rapid inhibition of the growth of *C. vulgaris* after exposure to phenanthrene for 72 h might have been related to the lipophilicity of phenanthrene, even when the concentration was only 1 μg L^−1^. When the exposure time extended to 168 h, the inhibition of the growth of *C. vulgaris* was alleviated at these two phenanthrene concentrations^20^. However, we observed that the growth of *C. vulgaris* was inhibited by 35% after acclimation for more than 10 days (Fig. 1). This may have partly resulted from the different culture methods applied: semi-continuous culture and batch culture were used for the present study and Calderón-Delgado’s study, respectively^20^. In addition, nutrient conditions and culture pH have been shown to modulate the toxicity of PAHs, with increased toxicity at lower pH^11^ and under enriched nutrient conditions^21^. Thus, the higher cell density and culture pH and lower nutrient concentrations in batch cultures of Calderón-Delgado’s study resulted in insignificant effects of phenanthrene^20^. For *S. costatum*, the phenanthrene concentration required to inhibit the growth rate by 50% was found to be 0.95 mg L^−1^ based on 72 h acute toxicity testing^22^. This was much higher than the concentration, at which cells did not grow (10 μg L^−1^) in the present study. This suggests that, in addition to acute toxicity, the chronic effects of phenanthrene should be considered when evaluating its toxicity to phytoplankton.

The low concentration of phenanthrene promoted the photosynthetic and electron transport rates of *S. costatum*, but inhibited these parameters with increasing concentrations of phenanthrene. It should be noted that the enhanced photosynthetic and electron transport rates were accompanied by the lower chlorophyll *a* content, indicating a higher light use efficiency at low phenanthrene concentration. This was also evidenced by the higher apparent photon transfer efficiency. Several studies have reported that low levels of environmental pollutants, such as PAHs, heavy metals, and pesticides, can stimulate the metabolic rates of plants and phytoplankton^23,24^. This phenomenon, in which a low dose of toxic substances is beneficial to organisms, is known as hormesis. Generally, a relatively high tolerance to low levels of PAHs was found in diatoms, and this tolerance decreases with increasing PAHs concentrations^25^. The underlying mechanism of hormesis may be that moderately increased levels of reactive oxygen species (ROS) caused by contaminants can activate beneficial signaling pathways to stimulate cellular processes^26^. The increase in cell density when hormetic stimulation occurs would then alleviate the negative effects of contaminants through dilution, as the contaminants would be segmented by more cells^27^. The hormesis caused by low concentrations of water pollutants was proposed to be one of supportive factors in the formation of phytoplankton blooms, which could be related to the vital role of cytochrome *b*_559_ in alleviating stresses imposed by pollutants^26^. The enhanced photosynthetic rates were not accompanied by increased growth in the present study. Growth is affected by different kinds of metabolic processes within cells and thus is usually a proxy of the comprehensive response. Unlike growth, photosynthesis is more sensitive to environmental changes with rapid responses. Thus, parameters related to photosynthesis should be measured in hormesis studies to provide a rapid and sensitive evaluation.

An imbalance between oxidation and antioxidation leads to the formation of damaging ROS, such as superoxide, hydrogen peroxide, and hydroxyl, causing oxidative stress to phytoplankton cells^28^. Enzymatic response is an antioxidant defense mechanism that plants have evolved to combat damage caused by ROS. SOD and CAT are important components of the first line defense system against ROS^29^. SOD is the earliest detoxifying enzyme and the most powerful antioxidant in cells, protecting cells from stressors. CAT catalyzes the degradation or reduction of hydrogen peroxide, thus completing the detoxification process stimulated by SOD^28^. In the present study, the SOD and CAT activity of *C. vulgaris* reached the maximum under the high phenanthrene concentration. In contrast, SOD was the main enzyme that acted to protect cells from ROS damage in *S. costatum*. It should be noted that the decrease of CAT activity at low and high phenanthrene concentrations found in the present study might have been caused by the damage exerted by excess ROS on cell viability. Additionally, the timing of enzyme analyses also influences the results, as shown by the increase and decrease of CAT activity of *S. costatum* after exposure to macrophytes pyrolysis bio-oil^30^. Thus, it is useful to test changes in the activity of the antioxidant enzymes with exposure time to give an overall picture of antioxidant defense mechanism.

Based on these results, phenanthrene at typical environmental concentrations could have detrimental effects on the growth and photosynthetic performance of phytoplankton via chronic effects. The different tolerance of phytoplankton species to phenanthrene toxicity would lead to changes in their relative abundances and community structures. Thus, phenanthrene contamination could significantly alter the ecological functions of marine ecosystems, even at concentrations as low as 1 μg L^−1^. Thus, it is important to consider both the acute and chronic responses of phytoplankton to contaminants, especially in coastal waters susceptible to PAH contamination.

## Methods

### Culture conditions

The green algae *C. vulgaris* (MASCC-0008) was obtained from the Marine Algae Stock Culture Collection Center (MASCC, Institute of Oceanology, Chinese Academy of Sciences), and the diatom *S. costatum* was originally isolated from the coastal waters of Gaogong Island, Jiangsu Province, China. Artificial seawater was used to culture cells after adding nutrients according to the recipe for Aquil medium recipe^31^. Culture temperature was maintained at 25 ℃ and cells were cultured in 500-mL polycarbonate bottles. The culture temperature was in the optimum growth temperature range according to previous studies^32,33^. The light intensity of 150 μmol photons m^−2^ s^−1^ with a 12:12 h light and dark cycle was set in a plant growth chamber.

### Experiment setup

The *C. vulgaris* and *S. costatum* cells were cultured in artificial seawater with three phenanthrene concentrations (0, 1, and 10 μg L^−1^ for *C. vulgaris*; 0, 1, and 5 μg L^−1^ for *S. costatum*, hereafter termed the control, low, and high concentrations) for at least 10 generations (8–12 days) before sampling and measuring. In the preliminary experiment, the *S. costatum* could not survive at the concentration of 10 μg L^−1^, so we adjusted the high concentration to 5 μg L^−1^. The stock solution (1 mg mL^−1^) was obtained by dissolving phenanthrene in dimethyl sulfoxide (DMSO). For the control treatment, the same volume of DMSO was added to the culture. Cultures were diluted every 3–4 days and the initial cell density was 50 cells per mL, and cell density was controlled below 1 × 10^5^ cells per mL before dilution. Three independent cultures were used for each treatment, and different treatments were cultured in one growth chamber to avoid artefacts.

### Specific growth rate

Samples for cell density measurement were retained and fixed with 10 μL Lugol’s solution. Cell density was measured with a plankton counting chamber using an optical microscope. The equation: µ = (lnN_1_ − lnN_0_)/(t_1_ − t_0_) was used to calculate the specific growth rate, where N_1_ and N_0_ represent cell densities at t_1_ and t_0_, respectively.

### Chlorophyll *a* and carotenoid contents

Cells for the determination of pigment contents were filtered onto GF/F filters (25 mm) and then extracted overnight in 5 mL absolute methanol at 4 °C in darkness.

After centrifugation for 10 min at 5000 *g*, the absorption values of supernatant were read by a spectrophotometer. The concentration of chlorophyll *a* was determined by the equation given by Ritchie^34^:

$${S. costatum\text{: }} {\text{Chl }a \text{ }}(\upmu {\text{g mL}}^{{ - {1}}} )\, = \,{13}.{2654}\, \times \,({\text{A}}_{665} {-}{\text{A}}_{750} ) \, {-}{2}.{6839}\, \times \,({\text{A}}_{632} {-}{\text{A}}_{750} );$$

$${C. vulgaris\text{: }} {\text{Chl }a \text{ }}(\upmu {\text{g mL}}^{{ - {1}}} )\, = \,{16}.{5169}\, \times \,({\text{A}}_{665} {-}{\text{A}}_{750} ) \, {-}{8}.{0962}\, \times \,({\text{A}}_{652} {-}{\text{A}}_{750} );$$

The concentration of carotenoid was calculated according to the equation of Strickland and Parsons^35^:

$${\text{Carotenoid }}(\upmu {\text{g mL}}^{{ - {1}}} )\, = \,{7}.{6}\, \times \,((A_{480} {-}A_{750} ) \, {-}1.49\, \times \,\left( {A_{510} {-}A_{750} )} \right),$$

in which A_x_ indicates the absorbance at wavelength x.

### Rapid light curve

The rapid light curve (RLC) was determined using an AquaPen-C fluorometer (AP-C100, Photon Systems Instruments). Samples were incubated at 150 μmol photons m^−2^ s^−1^ and 25 ℃ for 10 min before measurements. Six light intensities (10, 20, 50, 100, 300, and 500 μmol photons m^−2^ s^−1^) were set in the RLC measurements. Values of rETR were calculated according to rETR = PAR × Yield × 0.5, in which the yield represents the effective quantum yield of PSII, the PAR indicates the intensity of the actinic light level (μmol photons m^−2^ s^−1^), the factor 0.5 is the proportion of all absorbed quanta that PSII receives. RLCs data were fitted to the model: rETR = PAR/(a × PAR^2^ + b × PAR + c). The apparent photon transfer efficiency (α), light saturation point (I_k_), and maximum relative electron transport rate (rETR_max_) were calculated from a, b, and c according to the equation of Eilers and Peeters^36^.

### Photosynthesis and dark respiration

The oxygen evolution rate was determined by a Clark-type oxygen electrode (Oxygraph, Hansatech). The light intensity was set at 150 μmol photons m^−2^ s^−1^ and temperature (25 ℃) was controlled by a thermostatic water bath. The oxygen electrode chamber was covered with aluminum foil to measure the oxygen consumption rates in darkness.

Cells were gently filtered (< 0.02 MPa) onto a cellulose acetate membrane (47 mm), and they were then resuspended into Tris-buffered medium (20 mmol L^−1^). The pH of Tris-buffered medium was pre-adjusted by sodium hydroxide and hydrochloric acid to their corresponding culture medium value. The oxygen evolution and consumption rates were obtained from the linear portion of the oxygen record (10 min per sample).

### Superoxide dismutase and catalase activity

Cells were gently filtered (< 0.02 MPa) onto 0.8-µm polycarbonate filters (25 mm) and washed into 1.5-mL centrifuge tubes. Cells were broken by sonication in an ice-water bath (0 °C), and the homogenized extract was centrifuged at 11,100 *g* (4 °C) for 10 min before the superoxide dismutase (SOD) and catalase (CAT) activity were tested with SOD Assay Kit (A001-3, Jiancheng) and CAT Assay Kit (A007-1-1, Jiancheng), respectively.

### Statistical analysis

Triplicate cultures were used for each concentration, and the data were expressed as the mean value of triplicate cultures ± standard deviation (SD). One-way ANOVA and Tukey tests were used to analyze significant differences in parameters among concentrations.

## Data Availability

The data are available from the corresponding author on reasonable request.
